# A “Double-Locked” and Enzyme/pH-Activated Theranostic Agent for Accurate Tumor Imaging and Therapy

**DOI:** 10.3390/molecules27020425

**Published:** 2022-01-10

**Authors:** Jia Luo, Zongyu Guan, Weijie Gao, Chen Wang, Zhongyuan Xu, Chi Meng, Yun Liu, Yuquan Zhang, Qingsong Guo, Yong Ling

**Affiliations:** 1 Department of Pharmacy, The Affiliated Hospital of Nantong University, Nantong 226001, China; tdfylj@163.com; 2 School of Pharmacy, Nantong University, Nantong 226001, China; gntu2020@163.com (W.G.); 18806298941@163.com (C.W.); xzy424052148@163.com (Z.X.); jsxzmc123456@163.com (C.M.); yy2923036726@163.com (Y.L.); 3 Department of Obstetrics and Gynecology, The Affiliated Hospital of Nantong University, Nantong 226001, China; zyntu2012@163.com; 4 Department of Hepatobiliary and Pancreatic Surgery, The Affiliated Hospital of Nantong University, Nantong 226001, China

**Keywords:** β-Carboline, nitroreductase, NTR/pH dual-responsive, HDAC inhibitor, theranostic agent

## Abstract

Theranostic agents for concurrent cancer therapy and diagnosis have begun attracting attention as a promising modality. However, accurate imaging and identification remains a great challenge for theranostic agents. Here, we designed and synthesized a novel theranostic agent **H6M** based on the “double-locked” strategy by introducing an electron-withdrawing nitro group into 1-position of a pH-responsive 3-amino-β-carboline and further covalently linking the hydroxamic acid group, a zinc-binding group (ZBG), to the 3-position of β-carboline to obtain histone deacetylase (HDAC) inhibitory effect for combined HDAC-targeted therapy. We found that **H6M** can be specifically reduced under overexpressed nitroreductase (NTR) to produce **H6AQ**, which emits bright fluorescence at low pH. Notably, **H6M** demonstrated a selective fluorescence imaging via successive reactions with NTR (first “key”) and pH (second “key”), and precisely identified tumor margins with a high S/N ratio to guide tumor resection. Finally, **H6M** exerted robust HDAC1/cancer cell inhibitory activities compared with a known HDAC inhibitor SAHA. Therefore, the NTR/pH-activated theranostic agent provided a novel tool for precise diagnosis and efficient tumor therapy.

## 1. Introduction

Cancer, a main cause of death globally [1], leads to over 14 million new cases and 8.8 million deaths annually worldwide [2]. Early, accurate cancer diagnosis and therapy are crucial to increasing cancer survival [3]. Of the copious diagnostic methods available, fluorescence imaging with fluorescent probes as contrasts is used for in vivo cancer diagnosis because it aids simple, fast, and real-time monitoring [4]. Indocyanine green (ICG) and methylene blue (MB), the only fluorescent agents authorized by the US Food and Drug Administration, can be utilized to directly detect or image tumors. However, their performance remains unsatisfactory because they are “always-on” probes that produce fluorescence despite their interaction with the target sites, leading to non-specificity and low tumor-to-normal tissue (T/N) ratio [5]. Because of these defects, activatable probes for fluorescent diagnosis are highly needed. The fluorescence of these probes can be switched on specifically via the tumor microenvironment (TME), thus providing a high T/N ratio suited for surgical and chemotherapeutic applications [6,7,8]. Furthermore, integrating the fluorescence “off−on” mode and therapeutic capabilities into one system to create a theranostic molecule was proposed by Funkhouser. This is a useful strategy for precise in vivo diagnosis and chemotherapeutic administration [9,10,11]. Accordingly, theranostic agents based on the TME have been reported [12]. However, fluorogenic theranostic conjugates responding to a single cancer-related biomarker demonstrated unsatisfactory background fluorescence interference [13]. Hence, contributing to the development of theranostic agents with high accuracy to broaden the impact of theranostic precision medications is essential.

In recent years, tumor occurrence, development, and metastasis has been found to be closely correlated with the TME [14]. Dysregulated pH, caused by abnormal metabolism in most tumor cells, is widely considered a key cancer biomarker [11,15,16]. In detail, cancer cells feature low extracellular pH (6.6–7.1) along with low endosome and lysosome pH (5.5–6.5) [12,17,18]. Over the past few years, theranostic agents exploiting the acid microenvironment have been reported for cancer therapy [19,20,21]. However, their nonspecific activation by low pH in stomach tissue or any other nontarget sites may cause false-positive signals to impede their further clinical applications. To overcome this problem, the addition of a second limitation factor might be an alternative approach. In addition to dysregulated pH, hypoxia is considered another common characteristic of the TME [22], which simultaneously causes the overexpression of nitroreductase (NTR) [23]. NTR is a type of flavin-containing enzyme with an ability to catalyze the reduction conversion from extensive nitroaromatic compounds to the corresponding amines utilizing β-nicotinamide adenine dinucleotide (NAD) or β-nicotinamide adenine dinucleotide phosphate (NADP) as an electron donor [24]. However, few studies thus far have reported theranostic agents based on the NTR/pH successive reaction. Therefore, designing such “double-locked” theranostic agents activated by NTR and pH holds great potential in accurate bioimaging and therapy applications.

**Figure 1:**
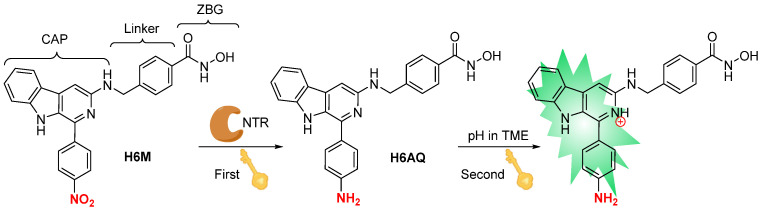
Design of “double-locked” and enzyme/pH-activated theranostic agent **H6M**.

Plants are a treasure trove of new drug discovery, where numerous researchers have presented a considerable amount of effort [25,26,27,28]. β-Carboline, a family of well-researched alkaloids containing a parent nucleus of tricyclic pyrido-[3,4-b]indole ring, isolated from *Peganum harmala L*., was reported to not only have potent anticancer activities but also demonstrate a significant amount of fluorescent emission due to its highly conjugated planar polyaromatic characteristic [29]. Our group previously developed several series of β-carboline derivatives and found that the 3-amino-β-carboline possessed greatly pH-dependent fluorescence for the first time [10]. Furthermore, in this work, an electron-withdrawing nitrophenyl group was introduced to 1-position of β-carboline to influence intramolecular charge transfer (ICT) [30]. The fluorescent properties of the compound were then noted to disappear immediately. Furthermore, we recently reported a β-carboline hydroxamic acid derivative, in which β-carboline (acting as a CAP group) is connected to the hydroxamic acid group. This derivative was aimed at adapting the tubular pocket of histone deacetylases (HDACs) and it demonstrated excellent antitumor activities. Thus, we embedded the active hydroxamic acid group of the HDAC inhibitor and the fluorescence quenching group nitrophenyl into the β-carboline so as to produce the novel theranostic agent **H6M** with dual fluorescence response of NTR and an acidic environment. Its nitro group may be selectively reduced to the amino compound **H6AQ** by the strongly expressed NTR (first “key”) in the TME, thereby releasing bright fluorescence under low pH (second “key”). This causes the formation of a “double-locked” mode with high accuracy but low background fluorescence. The expected scheme is illustrated in Figure 1. In addition, the intercalation of the β-carboline parent ring and the hydroxamic acid structure increased the compound’s inhibitory activity on tumor cells, so as to achieve precise diagnosis and treatment simultaneously.

## 2. Results and Discussion

### 2.1. Chemistry

**Scheme 1:**
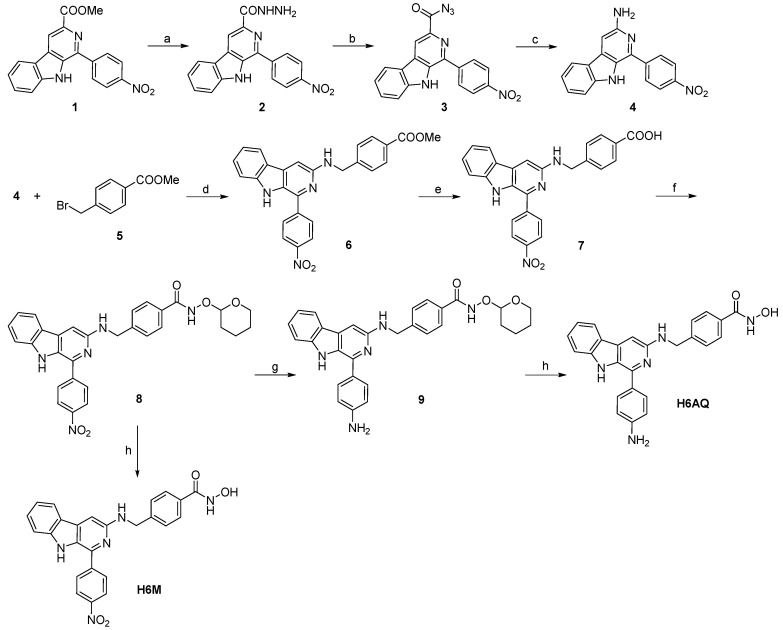
Synthetic route and reaction conditions: (a) N_2_H_4_·H_2_O, methanol, reflux, 4 h; (b) NaNO_2_, HCl (2 M), H_2_O, −5 °C, 1 h; (c) CH_3_COOH, H_2_O, 90 °C, 5 h (d) K_2_CO_3_, KI, CH_3_CN, r.t., 6h; (e) NaOH (2 M), methanol, reflux, 4 h; (f) *O*-(tetrahydro-2*H*-pyran-2-yl)hydroxylamine, HATU, DIPEA, DMF, r.t., 12 h; (g) Fe, NH_4_Cl, H_2_O, ethanol, 80 °C, 1 h; (h) methanolic HCl (2 M), r.t., 0.5 h.

To study the related properties, **H6M** and its nitro reduction product **H6AQ** were simply synthesized through a few reaction steps. As illustrated in Scheme 1, methyl 1-(4-nitrophenyl)-9*H*-pyrido[3,4-*b*]indole-3-carboxylate (compound **1**) was used as a starting material to react with hydrazine monohydrate and provide **2**, which was then mixed with sodium nitrite to provide acyl azide **3,** the acyl azide group of which was then converted to amino group via the Curtis rearrangement reaction to produce **4**. Next, **4** was reacted with *p*-bromobenzoate (compound **5**) in the presence of K_2_CO_3_ and KI to give the intermediate **6**, which was then hydrolyzed under aqueous NaOH to obtain **7**. After vacuum drying, condensation reaction between **7** and pyran hydroxylamine in the presence of 2-(7-azabenzotriazol-1-yl)-N,N,*N*’,*N*’-tetramethyluronium hexafluorophosphate (HATU) and N,N-diisopropylethylamine (DIPEA) was conducted to give **8**. Then, the nitro group was reduced using iron powder and NH_4_Cl to obtain **9**, which was finally deprotected in methanolic HCl to produce the amino compound **H6AQ**, and the designed compound **H6M** was directly deprotected from **8**. The final compounds **H6M** and **H6AQ** were purified to >95% purity via preparative chromatography and characterized by MS, ^1^H NMR, ^13^C NMR, and HRMS.

### 2.2. PH-Response of H6AQ

**Figure 2:**
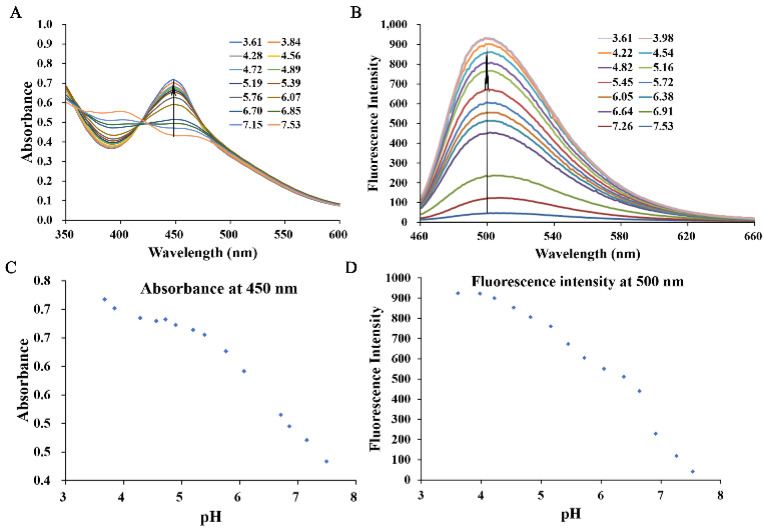
Spectra of normalized absorption (**A**) and fluorescence emission (**B**) for **H6AQ** (10 μM) at various pH values (3.61–7.53) in aqueous 10% (*v*/*v*) DMSO. Quantitative analysis of the absorption (**C**) and emission maximum (**D**) of **H6AQ** at various pH values (3.61–7.53) in aqueous 10% (*v*/*v*) DMSO (λ_ex_ = 450 nm).

Studies have found that the pH of tumor tissues is lower than that of normal tissues, and our group previously found that introducing the electron donating amino group in the 3-position of the β-carboline compound can obtain pH-sensitive fluorescence due to protonation/deprotonation under different pH levels. To confirm whether the reduced **H6M**, namely **H6AQ**, is pH responsive, we detected the shift of UV–vis spectroscopy as well as fluctuation of fluorescence emission spectroscopy within the range of pH 7.53–3.61. As shown in Figure 2, as the pH dropped from 7.53 to 3.61, the absorption peak of **H6AQ** gradually and bathochromically shifted from 398 to 450 nm with a >45-fold increase in fluorescence intensity at 500 nm (Figure 2A,B). The fluorescence intensity was noted to peak at pH 3.98 and remain unchanged with fluorescence quenching under basic conditions (Figure 2D). Therefore, **H6AQ** is suitable for monitoring the acidic environment of tumor tissues.

### 2.3. Optical Spectra of H6M and H6AQ

**Figure 3:**
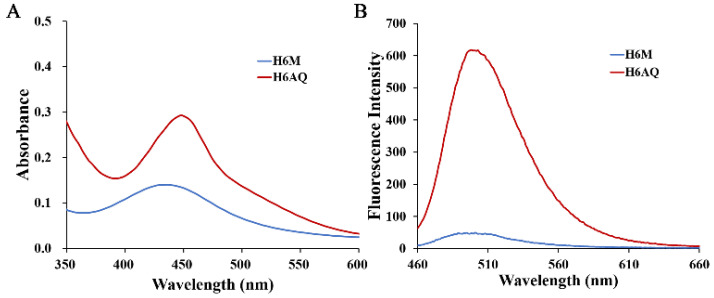
(**A**) Normalized absorption spectra of **H6M** (5 μM) and **H6AQ** (5 μM) and (**B**) fluorescence emission spectra of **H6M** (5 μM) and **H6AQ** (5 μM) in aqueous 10% (*v*/*v*) DMSO (λ_ex_ = 450 nm, pH = 3.98).

**Figure 4:**
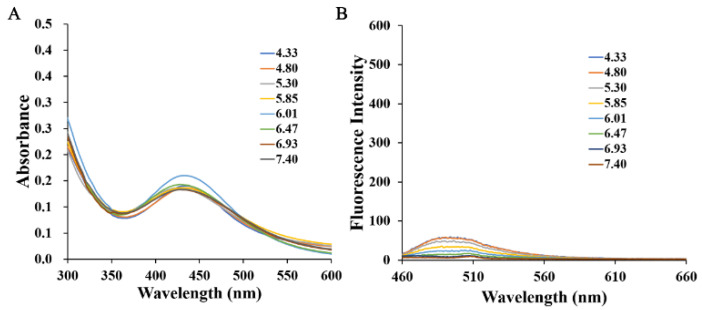
(**A**) Normalized absorption spectra of **H6M** (5 μM) at various pH values (4.33–7.40) and (**B**) fluorescence emission spectra of **H6M** (5 μM) in 10% DMSO aqueous (λ_ex_ = 450 nm).

The spectral properties of **H6M** and **H6AQ** were studied respectively. At pH 3.98, the absorption spectra of **H6M** was centered at 430 nm, whereas the reduced product **H6AQ** was centered at 450 nm (Figure 3A). Based on these, at excitation wavelength of 450 nm, **H6AQ** showed an emission maxima at 500 nm, whereas **H6M** remained nearly nonfluorescent (Figure 3B). Quantitative analysis revealed a >12-fold enhancement in fluorescence intensity from **H6M** to **H6AQ**, unambiguously indicating its “off–on” potential, which is in agreement with our assumption. Further analysis also demonstrated that **H6M** was independent of pH and could not be “unlocked” without the first “key” (Figure 4).

### 2.4. NTR/pH Dual-Response of H6M

**Figure 5:**
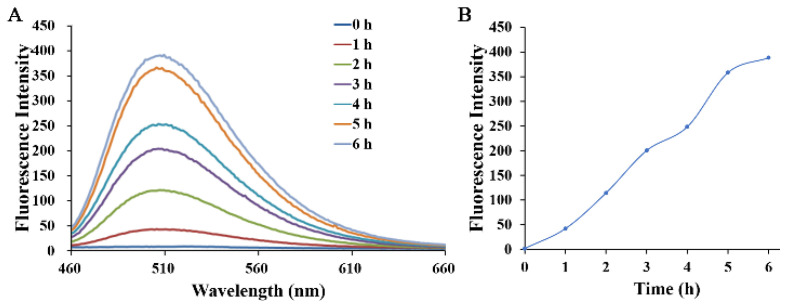
(**A**) Time-dependent fluorescence emission spectra of **H6M** (5 μM) incubated with NTR (10 µg/mL) and NADH (500 µM) in 10% DMSO aqueous with an excitation wavelength at 450 nm. (**B**) Fluorescence spectroscopic changes at 500 nm were monitored at 1-h intervals (pH = 4.00).

The results in this study thus far demonstrated that **H6AQ** with the amino group had pH-sensitive fluorescence but **H6M** with the nitro group was pH independent. Thus, to unlock the first lock, confirming whether **H6M** could be biologically reduced to **H6AQ** by NTR is necessary. We first performed an extracellular enzyme experiment in which we prepared an **H6M** solution at pH 4.00 in a mixed solvent (DMSO: H_2_O = 1:10) and then successively added NTR (10 µg/mL) and NADH (500 μM). Fluorescence changes in **H6M** treated with NTR and NADH were recorded at the timepoints of 0, 1, 2, 4, and 6 h. As shown in Figure 4, fluorescence enhancement was observed at 500 nm via excitation at 450 nm, and the fluorescence intensity remained unchanged after 6 h (Figure 5). The abovementioned spectral results implied that the “double-locked” **H6M** could be unlocked by NTR followed by pH for the accurate distinction of the tumor.

### 2.5. Selective Response of H6M to NTR and Low pH

**Figure 6:**
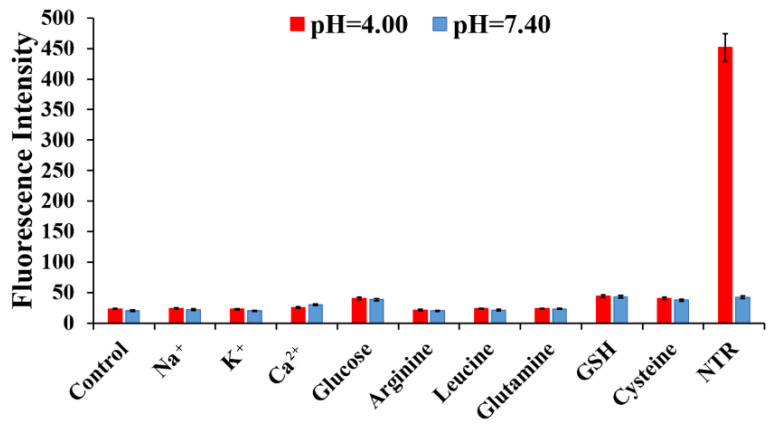
Fluorescence intensity of **H6M** (5 µM) in the presence of NTR (10 µg/mL) + NADH (500 µM) or other biologically relevant analytes (10 mM) at pH 4.00 and 7.40: (1) control, (2) Na^+^, (3) K^+^, (4) Ca^2+^, (5) glucose, (6) arginine, (7) leucine, (8) glutamine, (9) GSH, and (10) cysteine.

Although **H6M** can be reduced to **H6AQ** by NTR, the reduction of compound may be interrupted in vivo for an organism containing numerous components. Therefore, the fluorescence changes in **H6M** incubated with various analytes were recorded at pH 4.0 and 7.4. With excitation at 450 nm, **H6M** displayed almost no fluorescence increase with physiological ions (Na^+^, K^+^, and Ca^2+^), amino acids (arginine, leucine, glutamine, and cysteine), glucose, and GSH at both pH 4.0 and 7.4. On the addition of NTR, the fluorescence of the sensor solution significantly increased at 500 nm at pH 4.0 (Figure 6). These results confirmed high selectivity of **H6M** for NTR and low pH, providing a promising approach for cancer imaging in a complex microenvironment.

### 2.6. Cell Imaging

**Figure 7:**
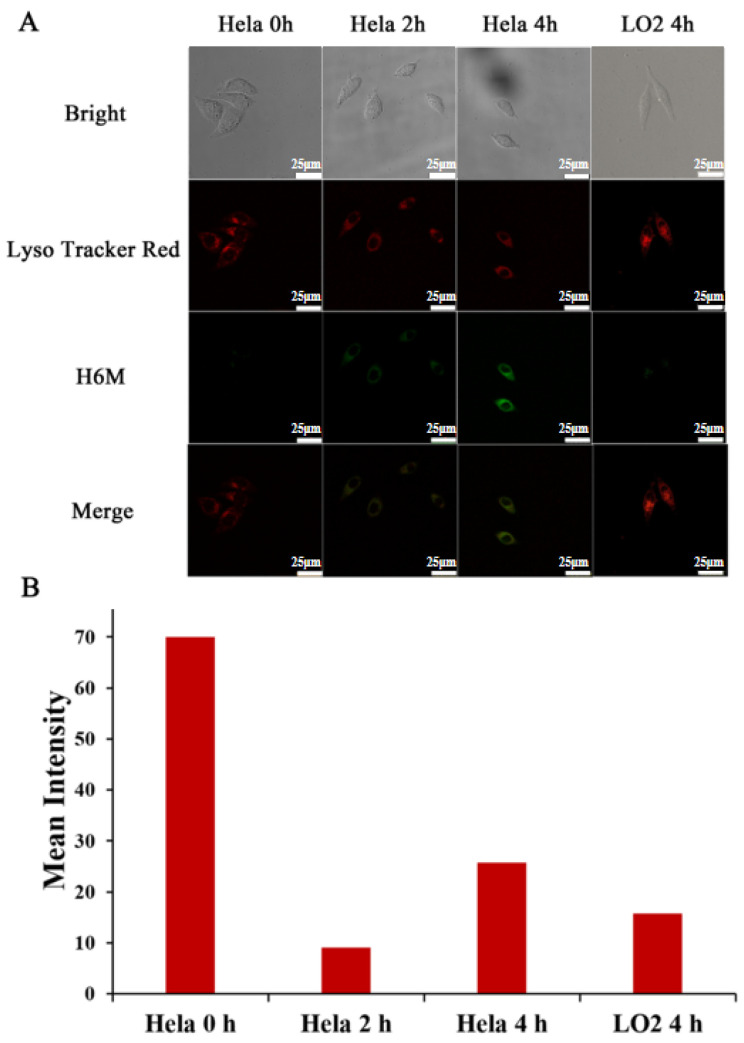
Intracellular fluorescence activation of **H6M**. (**A**) Confocal fluorescence images of HeLa and LO2 cells treated with **H6M** and LysoTracker red at different timepoints. Scale bar = 25 μm. (**B**) Mean fluorescence intensity of **H6M** in HeLa and LO2 cells were quantified at selected timepoints after incubation.

Based on the aforementioned results, the capability of **H6M** to image NTR in biological systems were evaluated in HeLa cells with NTR overexpression. The results revealed no fluorescence in the beginning because the nitro group produced a fluorescence masking effect (Figure 7). With time, green fluorescence became enhanced gradually for **H6M** that became progressively reduced to **H6AQ**. In contrast, consistently little to no fluorescence was observed in LO2 cells treated with **H6M**, suggesting that **H6M** selectively fluoresces in cancer cells with a signal-to-noise (S/N) ratio of >5.38.

### 2.7. Imaging-Guided Surgery of Tumors

**Figure 8:**
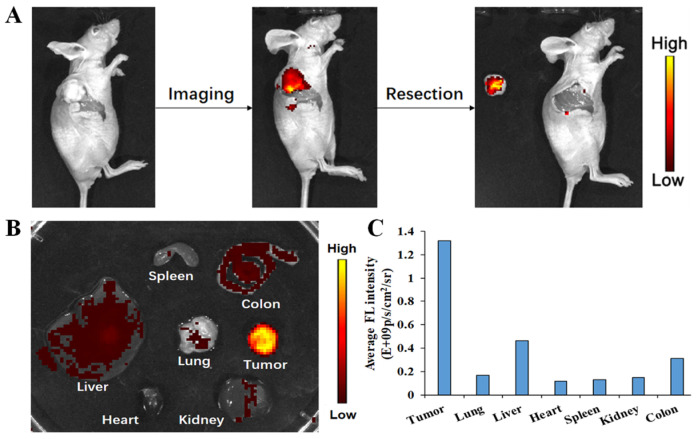
Fluorescence-guided location of tumors in BABL/c mice bearing HeLa xenograft tumors. (**A**) Image-guided resection after intravenous **H6M** injection in BABL/c mice bearing a HeLa xenograft tumor. (**B**) Ex vivo fluorescence imaging of dissected tumors and main organs of mice at 4 h after injection. (**C**) Quantitative analysis on ex vivo fluorescence imaging.

Complete resection of malignant tissue is vital during tumor surgery, but this can be challenging because fluorescent agents that can clearly identify tumor margins are lacking. Because it selectively aids the tumor imaging with high S/N ratio for the TME, **H6M** was further investigated for its ability to delineate tumors and guide tumor resection in HeLa tumor–bearing mice. As shown in Figure 8, after intravenous 40 mg/kg **H6M** administration, bright fluorescence was obvious at the tumor site, while the nontarget site was continually silenced. Guided by **H6M** fluorescence, cancer tissue was exposed and removed with a scalpel. To verify **H6M** specificity, mice were sacrificed. Their major organs and tumors were then collected and imaged on the Caliper IVIS Lumina II optical imaging system. As shown in Figure 8B,C, the fluorescent signal was significantly more intense in the tumors than in the major organs, with the T/N ratio ranging from 3.17 to 12.09. Taken together, these results demonstrated that **H6M** has great potential for identifying tumor margins and guiding cancer surgery in vivo.

### 2.8. Antitumor Activities of H6M and H6AQ

After evaluating the optical properties and application of **H6M** and **H6AQ**, we confirmed that **H6M** can be reduced to **H6AQ** to emit pH-sensitive fluorescence to achieve a “double-locked” effect for accurate imaging. Moreover, these two compounds are also HDAC inhibitors integrated with hydroxamic acid fragment. HDAC1 is an important HDAC subtype for cancer cell proliferation. Therefore, both the compounds may have potent enzyme/cancer cell inhibitory activity and therapeutic function. Thus, with harmine and the FDA-approved HDAC inhibitor SAHA as references, we tested the inhibitory activity of the two prepared compounds against HDAC1 and four human cancer cell lines (HCT116, HepG2, HeLa, and H1299; their IC_50_ values are displayed in Table 1). As predicted, harmine showed no marked activity against HDAC1, and its IC_50_ was >1000 nM. In contrast, the HDAC1 inhibitory activity of **H6M** and **H6AQ** was superior to that of the positive controls, indicated by excellent IC_50_ values (65 and 41 nM, respectively). These values were 2–3-fold lower than those of SAHA in HDAC1 inhibition. Similar to anti-HDAC1 activity, the anti-tumor cell proliferation activity of **H6M** and **H6AQ** clearly outperformed that of SAHA and harmine against all four cancer cell lines. Notably, compared with **H6M**, **H6AQ** had higher toxicity in all cancer cell lines, indicating that **H6M** may be more toxic upon reduction in cancer cells. In addition, **H6M** also showed lower cytotoxicity (IC_50_ = 12.98 μM) on normal human cell line LO2, which demonstrated that **H6M** had selective cytotoxicity to cancer cells.

## 3. Materials and Methods

### 3.1. General Information

All chemicals for synthesis were purchased from Aladdin Reagent (Shanghai, China) and used without further purification. ^1^H and ^13^C NMR were performed on a Bruker AV 400 M spectrometer using tetramethylsilane (TMS) as the internal standard. Fetal bovine serum (FBS) and Dulbecco’s modified Eagle medium (DMEM) were obtained from Thermo Fisher Scientific (Waltham, MA, USA) and Beijing BioDee Biotechnology (China), respectively. A trypsin-EDTA solution was obtained from Sigma-Aldrich (St. Louis, MO, USA). Tris-HCl buffer (pH 7.0) was obtained from Shanghai Yuanye Bio-Technology (Shanghai, China). The human hepatoma cell line HepG2, human cervical cancer cell line HeLa, human colorectal cancer cell line HCT116, and human non–small -cell lung cancer cell line H1299 were obtained from IBCB, CAS. UV−vis absorption and fluorescence spectra were attained via a UV1800PC spectrometer from Jinghua (China) and a fluorescence RF-5301PC spectrophotometer from Shimadzu (Japan), respectively. Compounds **2–4** were synthesized according to the literature [31], and NMR spectrum for compounds **6**, **8**, **H6M**, and **H6AQ** were attached in Supplementary Figures S2–S7.

### 3.2. Preparation of H6M

#### 3.2.1. Synthesis of Compound **6**

Compound **4** (97 mg, 0.32 mmol), KI (53 mg, 0.32 mmol), and K_2_CO_3_ (221 mg, 1.6 mmol) were mixed in a mixture comprising compound **5** (110 mg, 0.48 mmol) and CH_3_CN (3 mL). Then, the mixture was stirred at room temperature (r.t.), followed by evaporation under vacuum 6 h later and purification directly through flash column chromatography to provide compound **6** as a yellow solid (128 mg, yield: 88%). MS (ESI) *m/z =* 453 [M + H]^+^; ^1^H NMR (*d_6_*-DMSO, 400 MHz) δ: 11.90 (s, 1H, NH), 9.40 (m, 1H, NH), 8.87 (s, 1H, Ar-H), 8.42 (d, *J* = 7.9 Hz, 1H, Ar-H), 8.26 (m, 2H, 2Ar-H), 7.94 (d, *J* = 8.2 Hz, 2H, 2Ar-H), 7.70 (d, *J* = 8.2 Hz, 1H, Ar-H), 7.61 (m, 1H, Ar-H), 7.49 (m, 4H, 4Ar-H), 7.33 (m, 1H, Ar-H), 4.69 (d, *J* = 6.3 Hz, 2H, CH_2_), 3.84 (s, 3H, OCH_3_).

#### 3.2.2. Synthesis of Compound **8**

A solution of **6** (560 mg, 1.24 mmol) was prepared in 5 mL of methanol and added to 3 mL of 2 M aqueous NaOH solution. The reaction was refluxed at 65 °C for 4 h. Subsequently, the reaction was slowly added into ice-cold water, and pH was then adjusted to 7–8 to obtain a solid precipitate **7**, which was further reacted with *O*-(tetrahydro-2*H*-pyran-2-yl)hydroxylamine (145 mg, 1.24 mmol) in 6 mL of DMF and next mixed with to HATU (472 mg, 1.24 mmol) and DIPEA (160 mg, 1.24 mmol). The reaction was stirred at r.t. After 12 h, the reaction mixture was added to water with agitation, followed by extraction with 50 mL of DCM three times. The combined organic layer was dried over sodium sulfate and concentrated under vacuum. The crude residue was purified through flash column chromatography to obtain compound **8** as a yellow solid (288 mg, yield: 82%). MS (ESI) *m/z =* 538 [M+H]^+^; ^1^H NMR (CDCl_3_, 400 MHz) δ: 11.15 (s, 1H, NH), 8.87 (s, 1H, NH), 8.37 (m, 2H, Ar-H), 8.01 (m, 1H, Ar-H), 7.83 (d, 2H, *J* = 8.0Hz, Ar-H), 7.74 (d, 2H, *J* = 8.0Hz, Ar-H), 7.49-7.56 (m, 4H, NH, Ar-H), 7.33 (m, 1H, Ar-H), 7.19 (m, 1H, Ar-H), 7.03 (s, 2H, Ar-H), 5.08 (m, 1H, OCH), 4.65 (s, 2H, OCH_2_), 3.62-3.65 (m, 2H, OCH_2_), 1.80-1.88 (m, 6H, 3CH_2_).

#### 3.2.3. Synthesis of Compound **9**

A solution of **8** (360 mg, 0.67 mmol) in ethanol was mixed with iron powder (375 mg, 6.7 mmol) and 0.5 mL of saturated NH_4_Cl solution. The reaction was stirred vigorously at 80 °C for 1 h. The solution was filtered and then washed with ethanol to filter out insoluble substances. The solution was added into water, followed by extraction with 50 mL of DCM three times. The resulting organic layer was dried over sodium sulfate and evaporated under vacuum. Subsequently, the resulting residue was further purified through flash column chromatography to obtain compound **9** as a yellow solid (136 mg, yield: 40%). MS (ESI) *m/z =* 508 [M + H]^+^; ^1^H NMR (*d_6_*-DMSO, 400 MHz) δ: 10.95 (s, 1H, NH), 8.05 (d, 1H, *J* = 8.0Hz, Ar-H), 7.57–7.69 (m, 4H, NH, Ar-H), 7.36–7.47 (m, 5H, NH, CH=, Ar-H), 7.13 (m, 2H, Ar-H), 5.00 (m, 1H, CH), 3.68 (m, 2H, OCH_2_), 1.80-2.17 (m, 6H, 3CH_2_).

#### 3.2.4. Synthesis of H6AQ

Compound **9** (127 mg, 0.25 mmol) was added in a round flask containing 3 mL of 2 M methanolic HCl with stirring at r.t. for 30 min. Thereafter, the mixture was poured into water, followed by extraction with 50 mL of DCM three times. The collected organic layer was dried over sodium sulfate, followed by evaporation under vacuum to obtain crude residue. This residue was finally purified through flash column chromatography on silica gel to obtain **H6AQ** as a yellow solid (92 mg, yield: 87%). MS (ESI) *m/z =* 424 [M + H]^+^; ^1^H NMR (*d_6_*-DMSO, 400 MHz) δ: 11.03 (s, 1H, NH), 8.38 (m, 2H, NH, Ar-H), 8.22 (d, *J* = 8.7 Hz, 2H, 2Ar-H), 8.10 (m, 1H, Ar-H), 7.93 (d, *J* = 8.1 Hz, 2H, 2Ar-H), 7.59 (d, *J* = 8.1 Hz, 2H, 2Ar-H), 7.49 (d, *J* = 3.7 Hz, 2H, 2Ar-H), 7.31 (s, 1H, Ar-H), 7.15 (m,2H, 2Ar-H), 4.69 (s, 2H, NH_2_). ^13^C NMR (*d_6_*-DMSO, 100 MHz) δ: 164.7 (C=O), 152.6 (C), 147.0 (C), 145.5 (C), 145.4 (C), 143.3 (C), 135.4 (C), 134.6 (C), 132.2 (C), 129.5 (2CH), 128.8 (C), 127.8 (C), 127.7 (2CH), 127.3 (2CH), 124.2 (2CH), 122.8 (CH), 121.0 (CH), 119.0 (CH), 112.5 (CH), 98.4 (CH), 45.7 (CH_2_). HRMS (ESI): m/z calcd for C_25_H_22_N_5_O_2_: 424.1758; found: 424.1773.

#### 3.2.5. Synthesis of H6M

Compound **8** (108 mg, 0.20 mmol) was added in a round flask containing 3 mL of methanolic HCl (2 M). Then, the mixture was stirred at r.t. for 30 min. Thereafter, the mixture was poured into water, followed by extraction with 50 mL of DCM three times. The collected organic layer was dried over sodium sulfate and evaporated under vacuum to obtain crude residue, which was finally purified through flash column chromatography to obtain **H6M** as a yellow solid (80 mg, yield: 88%). MS (ESI) *m/z =* 454 [M+H]^+^; ^1^H NMR (*d_6_*-DMSO, 400 MHz) δ: 11.90 (s, 1H, NH), 9.42 (m, 1H, NH), 8.87 (s, 1H, Ar-H), 8.43 (d, *J* = 7.9 Hz, 1H, Ar-H), 8.26 (m, 2H, 2Ar-H), 8.06 (d, *J* = 8.2 Hz, 1H, Ar-H), 7.84 (d, *J* = 8.2 Hz, 1H, Ar-H), 7.70 (d, *J* = 8.2 Hz, 1H, Ar-H), 7.63 (m, 2H, 2Ar-H), 7.49 (m, 3H, 3Ar-H), 7.33 (m, 1H, Ar-H), 4.71 (m, 2H, CH_2_). ^13^C NMR(*d_6_*-DMSO, 100MHz) δ: 164.7 (C=O), 153.3 (2CH), 152.3 (C), 145.0 (C), 143.0 (C), 138.7 (C), 138.0 (C), 134.8 (C), 133.6 (C), 131.8 (C), 128.5 (2CH), 127.9 (2CH), 127.4 (C), 127.2 (C), 122.4 (CH), 121.2 (CH), 118.8 (CH), 112.5 (CH), 105.9 (2CH), 96.0 (CH), 45.8 (CH_2_). HRMS (ESI): m/z calcd for C_25_H_20_N_5_O_4_: 454.1515; found: 453.1501.

### 3.3. UV–Vis Absorption Spectra

All absorption measurement experiments were performed in **H6M** or **H6AQ** solution in DMSO/H_2_O solution (1:10, *v*/*v*) with a certain pH ranging from 7.53 to 3.61, and the absorption data were collected at r.t. on a UV-2450 spectrophotometer. The pH value of solution was adjusted by adding aqueous solutions of HCl or NaOH.

### 3.4. Fluorescence Spectra

Next, we prepared solutions of **H6M** or **H6AQ** in DMSO/H_2_O solution (1:10, *v/v*) with a certain pH ranging from 7.53 to 3.61. All emission spectra were performed on an RF-5301 fluorophotometer at r.t. with excitation at 450 nm. The pH value of solution was adjusted by adding aqueous solutions of HCl or NaOH.

### 3.5. Cell Culture

Cell lines of human hepatoma (HepG2), human cervical cancer (HeLa), human colorectal cancer (HCT116), human non–small -cell lung cancer (H1299), and human normal liver (LO2) were separately maintained in the DMEM/RMPI 1640 supplemented with 10% FBS (Invitrogen, Waltham, MA, USA) incubated at 37 °C in a humidified atmosphere containing 5% CO_2_.

### 3.6. Cytotoxicity Studies

The cytotoxicity of **H6M** and **H6AQ** in HepG2, HeLa, HCT116, and H1299 cells was determined using the 3-(4,5-dimethylthiazol-2-yl)-2,5-diphenyltetrazolium bromide (MTT) assay. Here, 0.25% trypsin was used to harvest exponentially growing monolayer cells. Single-cell suspension at a density of 1.0 × 10^4^ cells/well in 100 μL of cell culture medium were seeded into 96-well plates. After incubation overnight, **H6M**, **H6AQ**, SAHA, and harmine in DMEM/RMPI was added individually. After 72 h, these cells were washed with PBS and incubated with 0.5 mg/mL MTT, followed by incubation at 37 °C for another 3 h. We reported the detailed process in a previous study [10].

### 3.7. Evaluation of Selective Fluorescence

Next, 5 μM of **H6M** with NTR (10 µg/mL) + NADH (500 μM) and various physiological analytes (Na^+^, K^+^, Ca^2+^, glucose, Arginine, Leucine, Glutamine, GSH, Cystine) were prepared in deionized water with DMSO as cosolvent (DMSO: H_2_O = 1:10, pH 4.00 or 7.40). All fluorescent data were collected from 460 to 660 nm with excitation at 450 nm.

### 3.8. Intracellular Fluorescence Activation

Hela and LO2 cells were planted into 35-mm glass bottom cell culture dishes, respectively, at a density of 2 × 10^5^ cells/well and incubated in 2 mL of DMEM supplemented with H6M (1 μM) and LysoTracker red DND-99 (1 μM) for 15, 30, and 60 min. The fluorescence images were achieved on a confocal microscope (Leica TCS SP5). For H6M and LysoTracker red, excitation wavelength was 450 and 561 nm laser, respectively, and the filter was set at 480–530 and 580–630 nm, respectively.

### 3.9. Animals and Tumor Model

Six-week-old female BALB/c nude mice used here for animal experiments were authorized by the Animal Research and Care Committee of Nantong University (S20210507-012). They were bought from the Model Animal Research Center Affiliated to Nanjing University (Nanjing, China). These mice were administered a subcutaneous injection of 1 × 10^7^ HeLa cells in 200 µL of PBS to create a xenograft transplantation tumor model.

### 3.10. In Vivo Fluorescence Imaging and Imaging-Guided Surgery

The mice bearing HeLa tumors were used for in vivo fluorescence imaging. When the tumor grew to a size of about 300 mm^3^, **H6M** (40 mg/kg) was injected through their tail vein. Next, a Caliper IVIS Lumina II was used to image mice at 4 h after injection with an excitation and emission wavelengths at 450 and 500 nm, respectively. To evaluate imaging-guided surgery, subcutaneous tumor was exposed and resected under the guidance of fluorescence. Then, the mice were sacrificed, and their main organs, including the heart, liver, spleen, lung, kidney, and colon, were also collected and imaged using the above fluorescence imaging system.

### 3.11. Stability of H6M in Rat Plasma

**H6M** (100 μg/mL) standard solution was added to 30% rat plasma in PBS (0.5% DMSO), vortexed, and shaken to mix. Then, the mixture was placed at 37 °C constant temperature for appropriate times (0, 0.5, 1.0, 2.0, 4.0, 8.0, 12.0, and 24.0 h) in the shaking box. After vortexing at 0 °C for 2 min, the centrifuge was handled at 12,000 rpm/min for 5 min to gather the supernatant. The metabolic stability of **H6M** in rat plasma was measured and recorded by HPLC (Figure S1).

## 4. Conclusions

In conclusion, the “double-locked” and enzyme/pH-activated theranostic agent **H6M** was designed and synthesized in this study. **H6M** can be specifically reduced to **H6AQ** with an amino group by overexpressed NTR in the TME, and this yields a highly tumor-specific and pH-sensitive fluorescence signal with greatly reduced background signal in tumor cells. Both **H6M** and **H6AQ** were noted to have higher HDAC1 and tumor cell inhibitory abilities than the positive control SAHA. Furthermore, confocal fluorescence imaging demonstrated that **H6M** can selectively fluoresce in HeLa cells with high NTR expression, which led to successful imaging-guided tumor surgery in vivo. These results suggested that **H6M** integrated highly selective tumor diagnosis and efficient chemotherapy may be a useful tool for clinical accurate cancer therapy.

## Figures and Tables

**Table 1:** IC_50_ Values of **H6M** and **H6AQ** against HDAC1 and four cancer cell lines.

Compd	In Vitro Antiproliferative Activity (IC_50_, μM) *^a^*				HDAC1 Inhibition (IC_50_, nM) *^a^*
	HeLa	HepG2	HCT116	H1299	
SAHA	18.54 ± 0.32	5.95 ± 0.73	4.98 ± 0.56	7.01 ± 0.55	142 ± 18
Harmine	42.56 ± 4.15	53.8 ± 5.50	43.7 ± 3.85	ND *^b^*	>1000
**H6M**	3.59 ± 0.34	3.55 ± 0.18	3.23 ± 0.14	2.57 ± 0.22	65 ± 6
**H6AQ**	2.08 ± 0.30	2.58 ± 0.26	1.87 ± 0.21	1.69 ± 0.31	41 ± 5

*^a^* Each cell was treated with tested compounds for 72 h, and their cytotoxicity were determined by the MTT assay. Mean ± SD, *n* = 3. *^b^* Not detected.

## Data Availability

Not applicable.

## Supplementary Materials

Figure S1: Stability measurements of **H6M** incubated in Rat plasma at 37 °C and analyzed by HPLC at different times. The y-axis shows the relative concentration of the integrated peak areas of **H6M**. Figure S2: The ^1^H NMR spectra of compound **6**. Figure S3: The ^1^H NMR spectra of compound ^8^. Figure S4: The ^1^H NMR spectra of **H6M**. Figure S5: The ^1^H NMR spectra of **H6AQ**. Figure S6: The ^13^C NMR spectra of **H6M**. Figure S7: The ^13^C NMR spectra of **H6AQ**. Figure S8: HPLC analysis of compound **H6M**. Figure S9: HPLC analysis of compound **H6AQ**.

## Sample Availability

Samples of the compounds are not available from the authors.
