# Supplementary material for: A “Double-Locked” and Enzyme/pH-Activated Theranostic Agent for Accurate Tumor Imaging and Therapy
Source: Molecules. 2022 Jan 10;27(2):425. doi: 10.3390/molecules27020425 (PMC8779152; doi:10.3390/molecules27020425)
Supplement: Supplementary file 1 [file molecules-27-00425-s001.zip › molecules-1465414-supplementary.pdf]

Supplementary Information

A "Double-Locked" and Enzyme/pH-Activated Theranostic Agent for Accurate Tumor Imaging and Therapy

Jia Luo [1,2,†], Zongyu Guan [3,†], Weijie Gao [2,†], Chen Wang [2], Zhongyuan Xu [2], Chi Meng [2], Yun Liu [2],
Yuquan Zhang [3,*], Qingsong Guo [4,*], and Yong Ling [1,2,*]

[1] Department of Pharmacy, The Affiliated Hospital of Nantong University, Nantong 226001, China; tdfylj@163.com
[2] School of Pharmacy, Nantong University, Nantong 226001, China; gntu2020@163.com (W.G.); 18806298941@163.com (C.W.); xzy424052148@163.com (Z.X.); jsxzmc123456@163.com (C.M.); yy2923036726@163.com (Y.L.)
[3] Department of Obstetrics and Gynecology, The Affiliated Hospital of Nantong University,
Nantong 226001, China; zyntu2012@163.com
[4] Department of Hepatobiliary and Pancreatic Surgery, The Affiliated Hospital of Nantong University,
Nantong 226001, China
* Correspondence: ntgglj@163.com (Q.G.); Lyyy111@ntu.edu.cn (Y.L.); Leadspring@163.com (Y.Z.)
† These authors contributed equally to this work.

1. Stability measurements……………………………………………….....................S1
2. ^{1}H NMR spectrum for compound **6**, **8**, **H6M**, and **H6AQ**……………………...S2
3. ^{13}C NMR spectrum for compound **H6M** and **H6AQ**……………………………S4
4. HPLC analysis of compound **H6M** and **H6AQ**…………………………………S4

1. Stability measurements

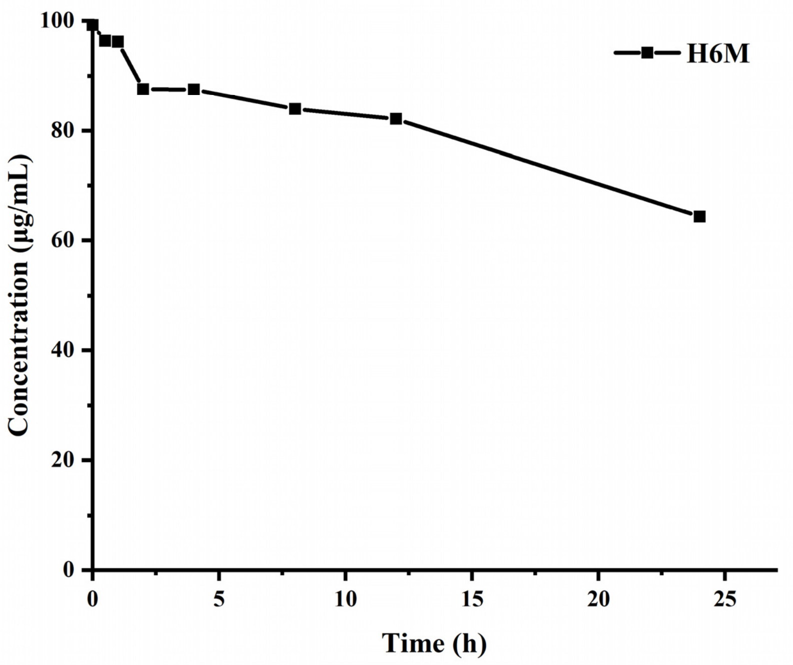

Figure S1. Stability measurements of **H6M** incubated in Rat plasma at 37 °C and analyzed by HPLC at different times. The y-axis shows the relative concentration of the integrated peak areas of **H6M**.

2. ^{1}H NMR spectrum for compound **6**, **8**, **H6M**, and **H6AQ**

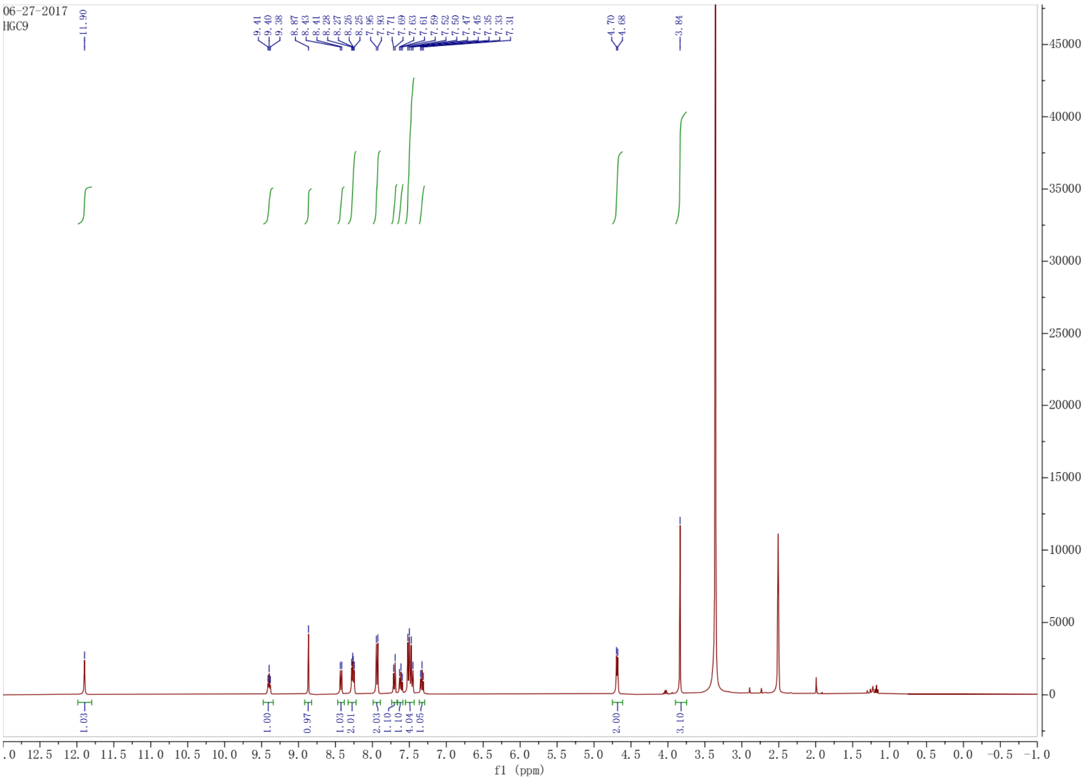

Figure S2. The ^{1}H NMR spectra of compound **6**

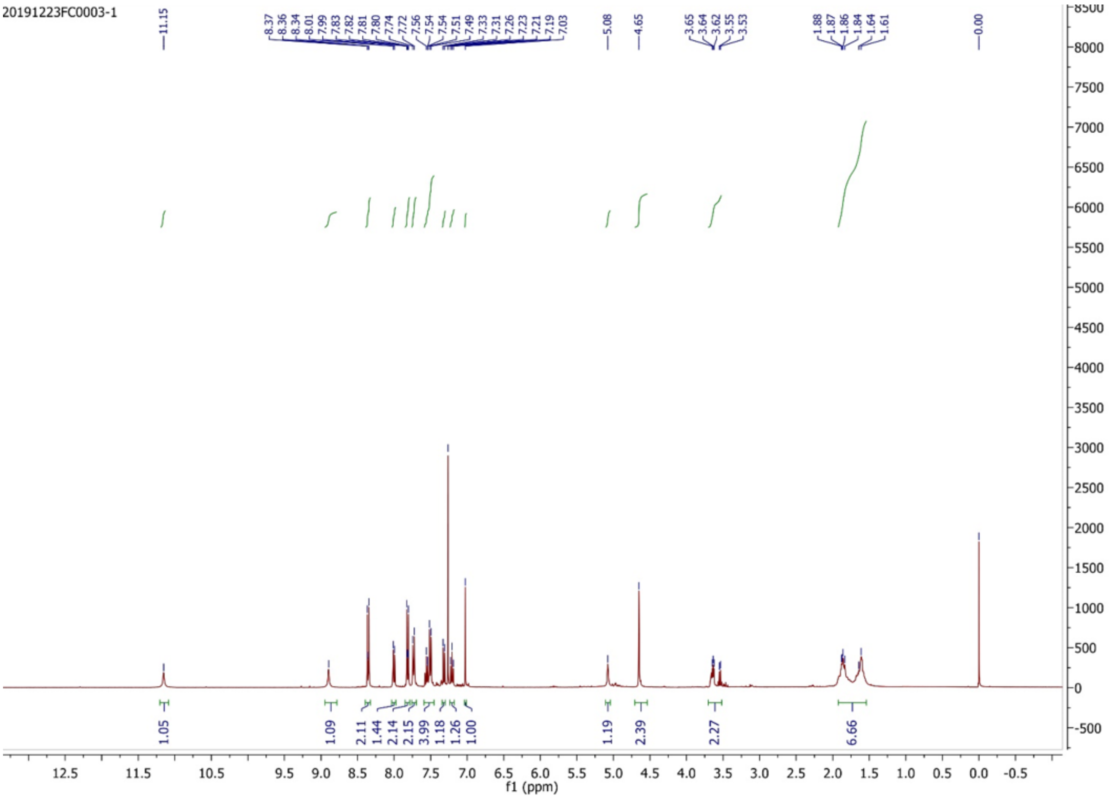

Figure S3. The ^{1}H NMR spectra of compound **8**

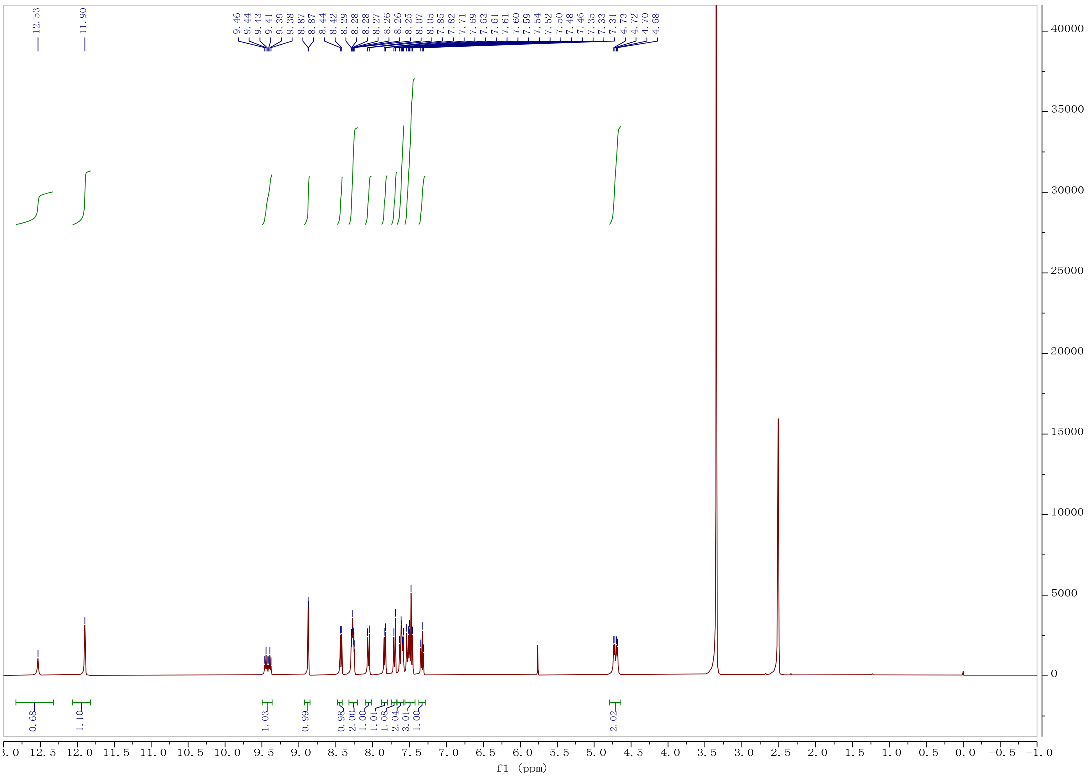

Figure S4. The ^{1}H NMR spectra of **H6M**

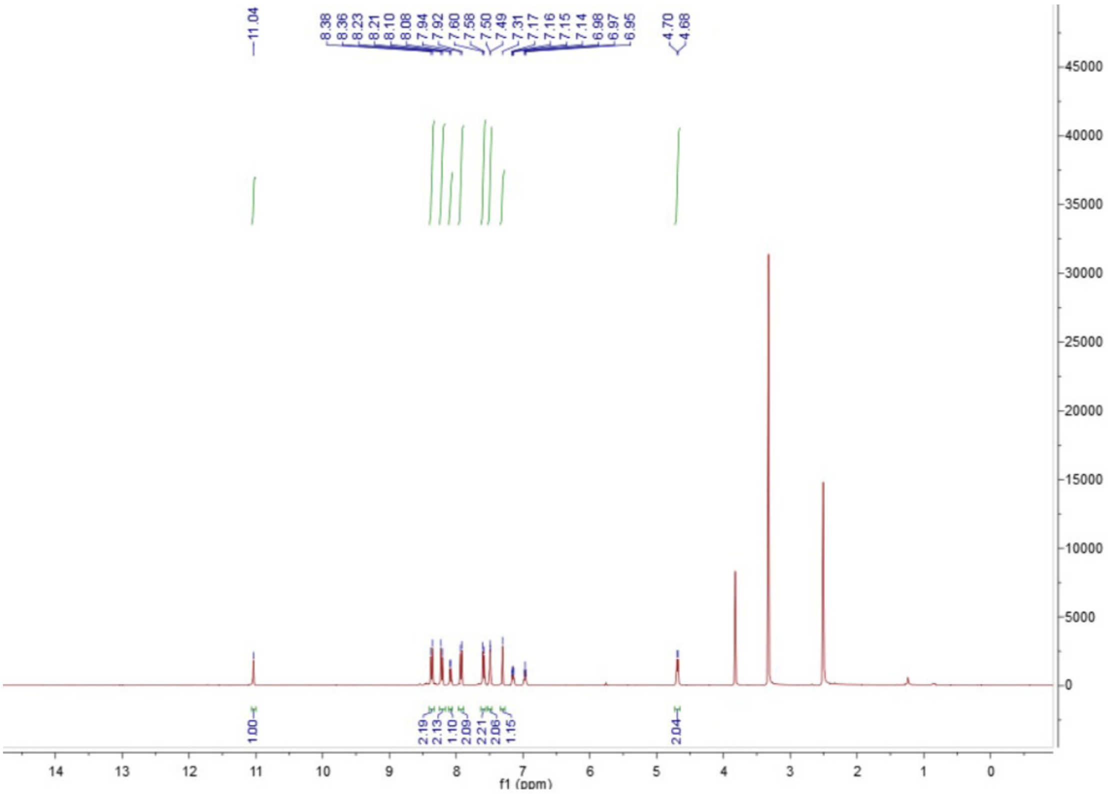

Figure S5. The ^{1}H NMR spectra of **H6AQ**

3. ^{13}C NMR spectrum for compound **H6M** and **H6AQ**

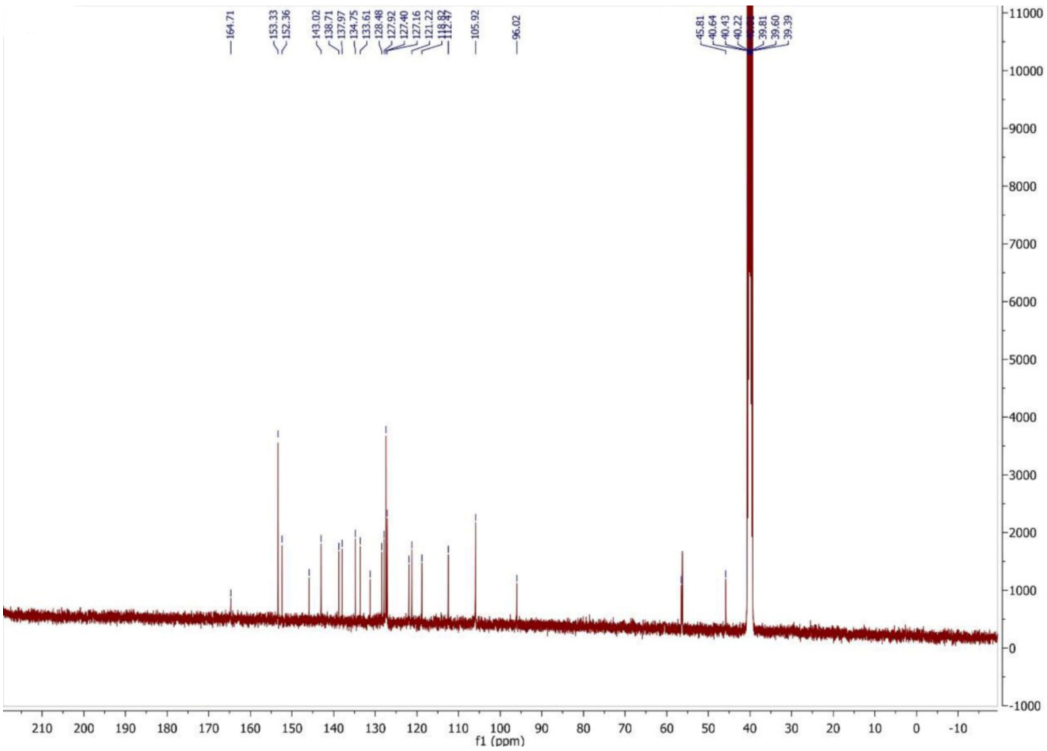

Figure S6. The ^{13}C NMR spectra of **H6M**

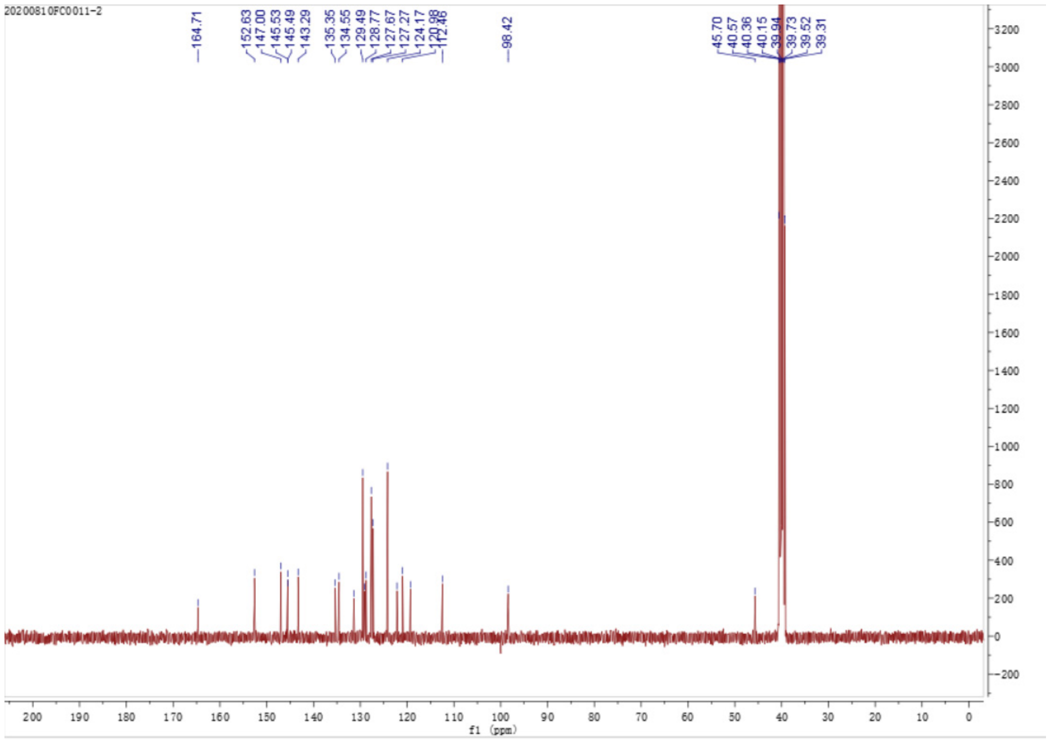

Figure S7. The ^{13}C NMR spectra of **H6AQ**

4. HPLC analysis of compounds H6M and H6AQ.

Method: 80% methanol aqueous solution, flow rate =1 ml/min, Agilent Eclipse Plus-C18 4.6*250 mm, 5 µm, temp 25 °C, wavelength 254 nm.

Compound **H6M**: Purity 97%.

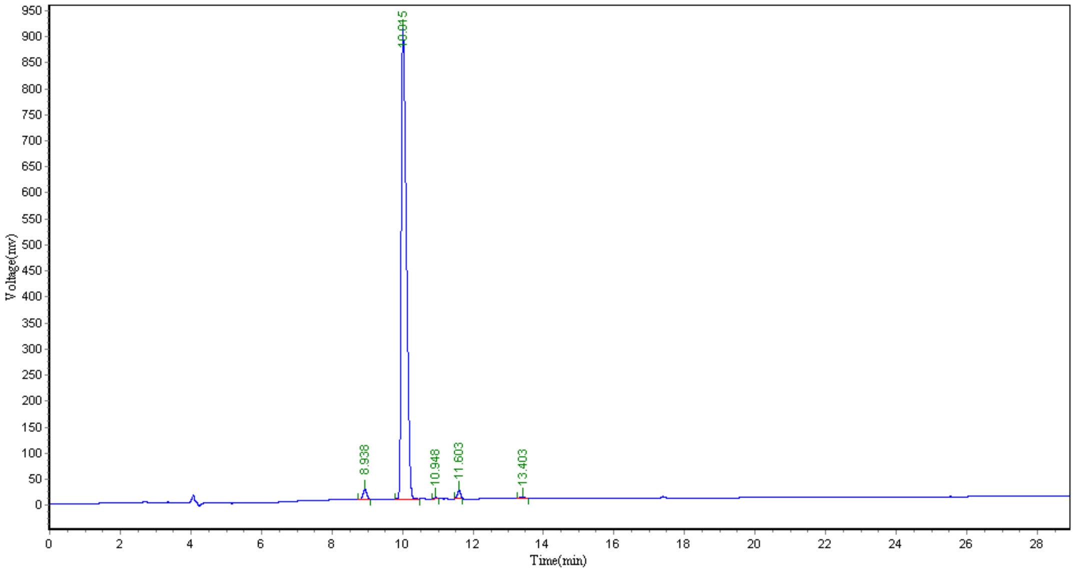

Figure S8. HPLC analysis of compound **H6M**

Compound **H6AQ**: Purity 99%.

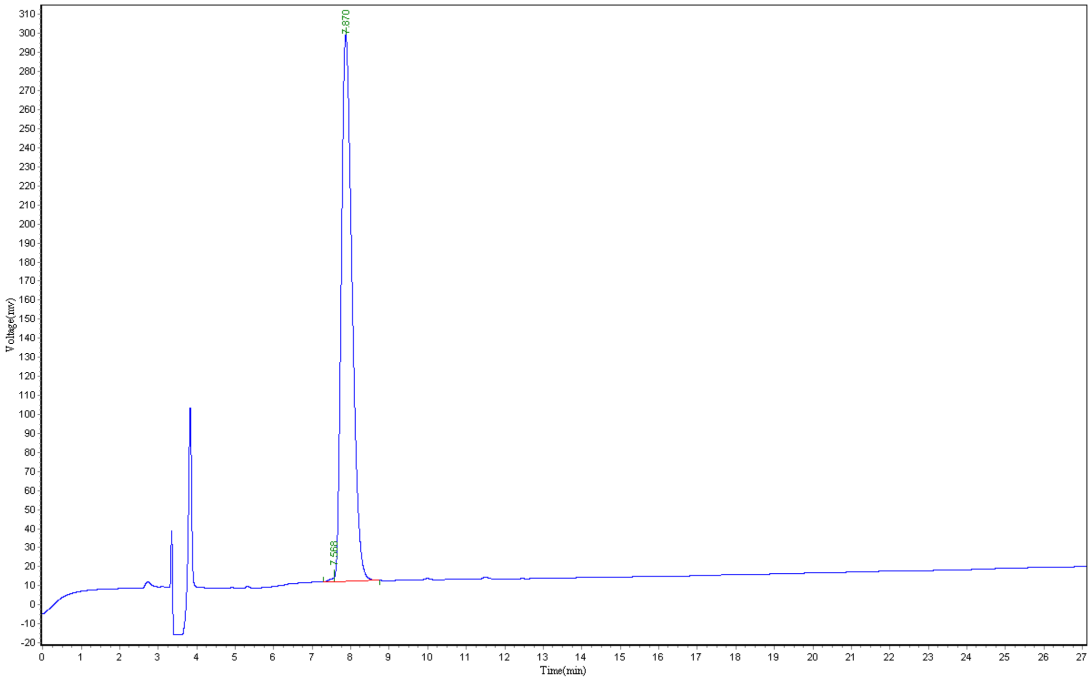

Figure S9. HPLC analysis of compound **H6AQ**
